# From Dysbiosis to Disease: How Vaginal Microbiota Shape Group B Streptococcus Pathogenesis

**DOI:** 10.1002/cbic.70573

**Published:** 2026-10-23

**Authors:** Julie A. Talbert, Steven D. Townsend

**Affiliations:** ^1^ Department of Chemistry Vanderbilt University Nashville Tennessee USA

**Keywords:** biology, disease, dysbiosis, lactobacillus, microbiology, pathogen, streptococcus, streptococcus agalactiae, virulence

## Abstract

Host–microbe interactions refer to the dynamic relationships between hosts, such as humans, and the microorganisms present on or inside them. These associations can be beneficial, like gut bacteria facilitating digestion, or harmful, such as pathogens leading to disease. This review focuses on the reproductive tract, specifically the vaginal microbiome. Typically, a healthy vaginal microbiome is dominated by *Lactobacillus* species, which help protect against infections by pathogens. Dysbiosis, or a microbial imbalance, allows pathogens to invade. One such pathogen is *Streptococcus agalactiae* (group B *Streptococcus*, GBS), which can colonize the vaginal microbiome and cause infections. GBS infection during pregnancy is particularly concerning and is a major cause of adverse pregnancy outcomes. This review will explore host–microbe interactions in the vaginal microbiome, with an emphasis on GBS infection, its virulence, and current treatment strategies.

## Graphical abstract

*Lactobacillus crispatus* plays a critical role in the vaginal microbiome. Limiting glucose shifts metabolism toward HMO consumption and amino acid metabolism. These actions enable alkali production and competition. Homeostasis, characterized by dense Lactobacilli colonization and suppression of pathogens, provides resistance to dysbiosis.

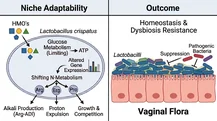

## 1 Common Host–Microbe Interactions in Humans

There are three main forms of symbiosis, which are defined as close, enduring interactions between a host organism and a microbe. Most of these associations are mutualistic, meaning that both parties gain benefits. Commensalism describes a relationship in which one species benefits without harming the other. Parasitism, or pathogenesis, occurs when the microbe gains an advantage at the host’s expense. Pathogens in humans are categorized as invasive species, opportunistic pathogens, or pathobionts. Opportunists are typically acquired externally and cause infections when the host’s defenses are compromised. Pathobionts are members of the native microbiota that are normally harmless but can cause invasive infections under specific genetic or environmental stressors.

Within each ecological microbiota, a variety of selective pressures are at play, such as temperature, pH, host immune responses, mechanical forces, oxygen availability, and nutrient sources. When focusing on the vagina, key selective pressures include the intense acidity, mucosal flow, and presence of host‐derived glycans as the sole nutrient. Overall, these diverse pressures determine the unique microbial composition of each ecological niche.

## 2 The Vaginal Microbiome

### 2.1 Lactobacillus Species Dominate Healthy Vaginal Microbiomes

The vaginal environment plays an essential role in maintaining female reproductive health from adolescence through menopause. Although it was largely overlooked in earlier research, our understanding of the vaginal microbiome and the surrounding reproductive tissues has grown considerably in recent times [1, 2]. In 2011, Ravel and Forney applied 16S rRNA gene sequencing to vaginal samples from 396 asymptomatic women across North America [3]. Based on these data, the microbial communities were categorized into five distinct groups, known as community state types (CSTs) (Table 1). CST I was characterized by the dominance of *Lactobacillus crispatus*, while groups II, III, and V were primarily made up of *Lactobacillus gasseri*, *Lactobacillus iners*, and *Lactobacillus jensenii*, respectively. Although dominance by *Lactobacillus* generally indicates a protective vaginal environment, the prevalence of *L. iners* often signifies a transitional phase, hinting at a potential shift toward dysbiosis [4, 5]. The last group, CST IV, displayed more diverse microbial profiles, with higher levels of anaerobic bacteria.

**TABLE 1:** Community state types in the vaginal microbiome.

CST	Dominant bacterial species	Protective or disruptive?	% of population
I	*L. crispatus*	Protective	~26.2
II	*L. gasseri*	Protective	~6.3
III	*L. iners*	Neutral, but can lead to disruptive	~34.1
IV	Diverse	Often disruptive	~25.0
V	*L. jensenii*	Protective	~5.3

Since this seminal study, groups have continued to refine the vaginal CST classifications, but the five CSTs in Table 1 are still the most commonly used [6, 7]. Advances in metagenomics and metatranscriptomics are deepening our understanding of these communities’ functional roles at the species level, shifting our focus from simply identifying species to exploring what these communities actually do [8, 9]. The selective pressures outlined in Section 1, such as acidity, dependence on glycogen metabolism, the predominantly anaerobic environment, and the physical barrier of the mucosa, collectively influence these functional profiles.

### 2.2 The Effects of the Menstrual Cycle on the Microbiota

Hormone fluctuations throughout the menstrual cycle impact the vaginal environment. Menstruation marks the beginning of the cycle, during which microbial communities experience notable changes, leading to heightened bacterial diversity (Figure 1) [10, 11, 12]. Most significantly, blood flow during menstruation leads to a more neutral pH, thereby negatively affecting lactobacilli that thrive in acidic conditions and promoting the colonization of opportunistic pathogens and dysbiosis‐associated bacteria [11, 13, 14, 15, 16, 17, 18].

**FIGURE 1:**
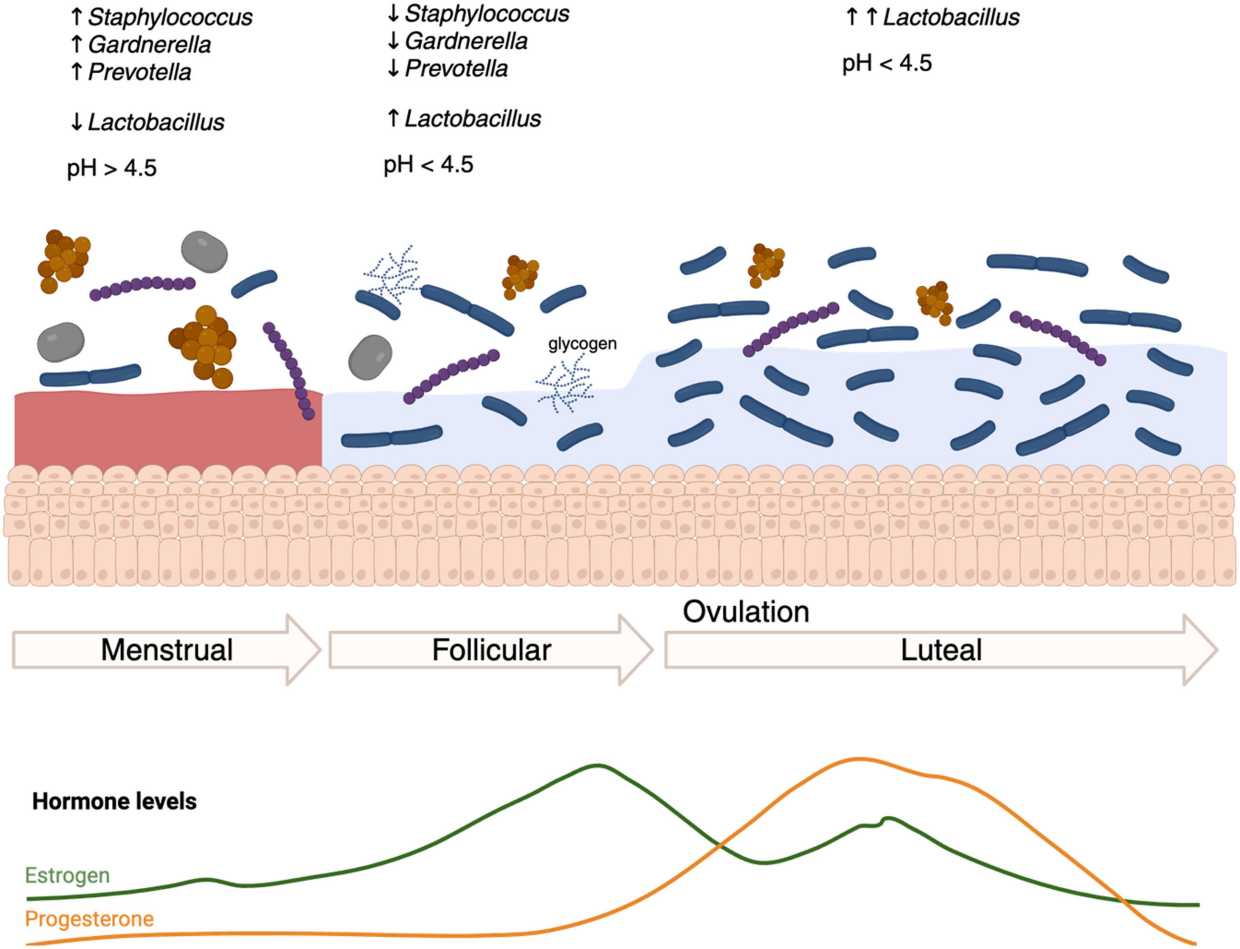
The menstrual cycle affects the vaginal microbial community. Increased blood flow during the menstrual cycle leads to decreased lactobacilli and increased diversity. The follicular phase is marked by increased glycogen, which supports *Lactobacillus* growth. Finally, the luteal phase is characterized by further increases in *Lactobacillus* and a thicker mucus layer.

During the follicular phase, pH returns to acidic and estrogen peaks [19, 20]. Increased estrogen promotes the maturation of the glycogen‐rich vaginal epithelium, in which glycogen is released as cells are regularly lysed [21, 22, 23]. Increased glycogen correlates with increasing *Lactobacillus* abundance, and the *Lactobacillus*‐dominated vaginal environment remains stable through subsequent phases until menstruation [11, 12, 15].

During the ovulatory and luteal phases, progesterone and estrogen levels fluctuate. These fluctuations regulate the physical properties of the mucosal barrier, a packed hydrogel network of mucin glycoproteins [20, 24, 25]. Mucins serve as substrates for bacterial attachment and as a nutrient source. Indeed, species of *Lactobacillus*, *Staphylococcus*, *Streptococcus agalactiae* (Group B *Streptococcus*, GBS), and *Enterococcus faecalis* bind mucins [26, 27, 28, 29, 30, 31, 32]. Species that use mucins as a nutrient source have evolved glycosidase activity that cleaves terminal residues from mucins for energy [33, 34]. Overall, this cyclical dysbiosis creates intermittent windows of vulnerability to pathogen invasion.

## 3 Mechanisms of Colonization Resistance: How Lactobacilli Defend the Vaginal Niche

### 3.1 Colonization Resistance in the Vaginal Environment

The healthy microbiome employs various strategies to defend its distinct niche (Figure 2). The vaginal microbiome primarily relies on lactic acid production as its strongest defense [14, 35, 37]. This acid keeps the pH between 3.5 and 4.5, making it hostile to most pathogens. Another key resistance mechanism is physical attachment, which promotes biofilm formation [38, 39]. Lactobacilli excel at binding to vaginal epithelial receptors, reducing the available sites for pathogens. Nutrient sequestration also plays a crucial role by starving invading microbes. Lactobacilli are known for their high metabolic activity; they quickly consume nutrients such as glycogen, amino acids, and metals, creating a nutrient‐poor environment [35, 40]. Consequently, pathogens must be evolutionarily equipped to compete with lactobacilli.

**FIGURE 2:**
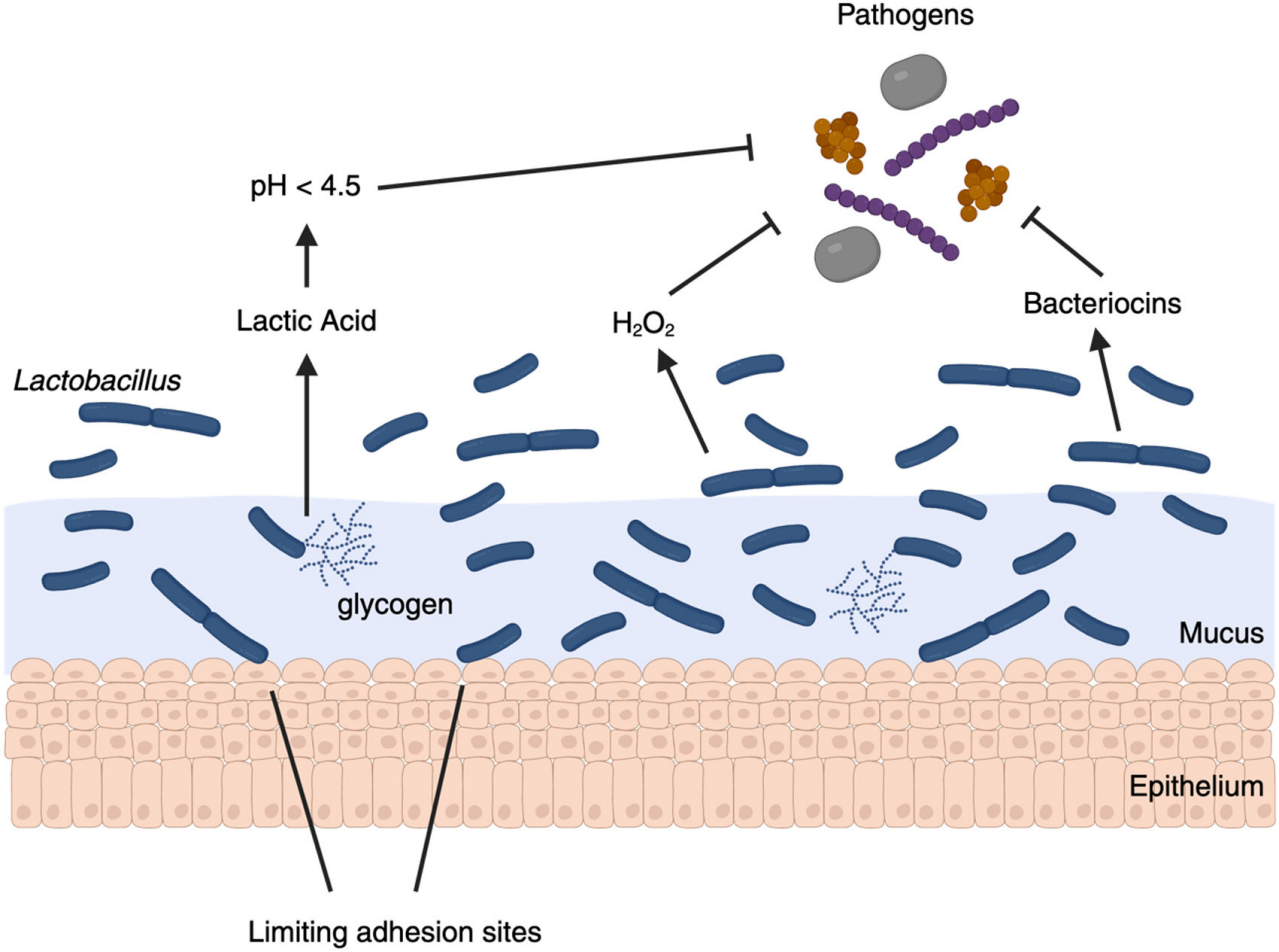
The various mechanisms by which vaginal lactobacilli defend their niche from pathogenic colonization.

For a more direct antimicrobial approach, common strategies include lactobacilli producing hydrogen peroxide (H_2_O_2_) and bacteriocins [41]. H_2_O_2_‐producing *Lactobacillus* spp. are typically found more often in healthy microbiotas than in dysbiotic states. However, the precise role of H_2_O_2_ in this environment remains debated, as discussed by Miko and Barakonyi [42]. Bacteriocins are small antimicrobial peptides (AMPs). Maldonado‐Barragán’s team discovered that gassericin E, produced by a vaginal strain of *L. gasseri*, exhibits antibacterial activity against various pathogens [43]. Specifically, Alajekwu and Ike extracted bacteriocin‐like inhibitory substances from seven vaginal *Lactobacillus* strains and found they were active against GBS [44]. Overall, a healthy vaginal microbiome employs various mechanisms to prevent pathogen colonization.

### 3.2 Vaginal Dysbiosis States and Key Pathogens

Microbial dysbiosis refers to an imbalance in the bacterial community composition, especially when the beneficial microbes that form mutualistic relationships with the host are diminished. In the vaginal microbiome, this imbalance is often marked by reduced *Lactobacillus* levels and increased microbial diversity. Table 2 lists common vaginal pathogens along with their clinical effects. Factors contributing to dysbiosis include intravaginal cleaning, sexual activity, smoking, use of antibiotics, hormonal changes, genetics, contraceptive use, and ethnic or socioeconomic differences [45, 46, 47, 48, 49, 50, 51].

**TABLE 2:** Common vaginal pathogens and associated clinical manifestations.

Category	Pathogen	Type	Trigger/entry	Clinical manifestations
Dysbiotic species	Various aerobic species	Bacteria	pH elevation, loss of *Lactobacillus*	Bacterial vaginosis (BV)
	*Candida albicans*	Fungus	Antibiotic use (microbial imbalance)	Vulvovaginal candidiasis (VVC)
Invasive	*Trichomonas vaginalis*	Protozoan	Sexual transmission, pH elevation	Sexually transmitted infection (STI)
	*Chlamydia trachomatis*	Bacterium	Sexual transmission, pH elevation	Sexually transmitted infection (STI)
	*Neisseria gonorrhoeae*	Bacterium	Sexual transmission, pH elevation	Sexually transmitted infection (STI)
Gastrointestinal migrants	*Escherichia coli*	Bacterium	Gut migration, pH elevation	Aerobic vaginitis (AV)
	*Staphylococcus aureus*	Bacterium	Gut/skin migration, pH elevation	Aerobic vaginitis (AV)
Pathobionts	*Streptococcus agalactiae*	Bacterium	Common resident	Adverse pregnancy outcomes

A decline in *Lactobacillus* levels correlates with an increase in vaginal pH, creating an environment less supportive of beneficial bacteria and more prone to pathogen growth. The most recognized form of dysbiosis is bacterial vaginosis, which involves a shift from *Lactobacillus* to anaerobic bacteria like *Gardnerella*, *Prevotella*, and *Fannyhessea* rather than a single infectious agent [52, 53]. Several sexually transmitted infections (STIs) are also linked to dysbiosis. For example, *Trichomonas vaginalis*, a parasitic protozoan, produces alkaline substances that raise the vaginal pH and eliminate lactobacilli [54]. Infection with *T. vaginalis* heightens the risk of preterm birth and human immunodeficiency virus (HIV) transmission. The most common bacterial STIs are caused by *Chlamydia trachomatis* and *Neisseria gonorrhoeae*, which can invade the reproductive tract in a dysbiotic environment, leading to pelvic inflammatory disease (PID) [55]. The long‐term effects of PID may include ectopic pregnancy or permanent infertility. Additionally, bacteria like *E. coli* or *S. aureus*, often originating from the gut microbiome, can cause aerobic vaginitis (AV), characterized by an overgrowth of aerobes [56].

Some dysbiotic conditions are not associated with elevated pH levels. For example, vulvovaginal candidiasis (VVC) is a common condition where the fungus *Candida albicans* colonizes the vaginal environment, causing severe inflammation [57]. *C. albicans* prefers acidic conditions, and its overgrowth is often stimulated by antibiotic use. Another well‐known vaginal pathogen, which is the main subject of this review, is *Streptococcus agalactiae* (group B *Streptococcus*, GBS). Like *C. albicans*, GBS survives in the acidic vaginal environment and is mainly associated with negative pregnancy outcomes.

## 4 Group B *Streptococcus*

### 4.1 Clinical Relevance of Streptococci

Streptococci are Gram‐positive cocci that are non‐motile, non‐spore‐forming and usually appear in pairs or chains. This genus plays a crucial role in human health, comprising over 50 known species that often reside in the respiratory, gastrointestinal, and genitourinary tracts [58]. *Streptococcus* are facultative or obligate anaerobes, capable of surviving in low‐oxygen environments. Within this genus, strains are classified using the Lancefield grouping, which identifies specific carbohydrate antigens on the cell wall. Additionally, streptococci are categorized by their hemolytic activity into three groups: beta (β), alpha (α), and gamma (γ). β‐hemolytic strains completely lyse red blood cells, α‐hemolytic strains partially lyse them, and γ‐hemolytic strains show no hemolysis.

Members of the *Streptococcus* genus cause infections in various human niches. *Streptococcus pyogenes*, or group A *Streptococcus* (GAS), is the leading cause of upper respiratory infections in humans and is best known for causing “strep throat.” *Streptococcus pneumoniae* is another common *Streptococcus* pathogen that is frequently associated with lower respiratory tract infections. Finally, GBS is predominantly a commensal organism but can cause invasive infection in immunocompromised individuals, including pregnant people and infants.

### 4.2 Group B *Streptococcus* Strain Classifiers

GBS is a *β*‐hemolytic, Gram‐positive pathobiont, and facultative anaerobe that can form robust biofilms. Following classification by hemolysis, GBS strains are differentiated by capsular polysaccharide (CPS) serotype using immunological assays that measure CPS antigenicity. Ten serotypes are currently recognized: Ia, Ib, and II–IX. Each CPS is composed of carbohydrate repeating units with characteristic monosaccharide composition, glycosidic linkages, branching patterns, and, in some serotypes, structural modifications such as acetylation and terminal sialylation. Although the serotypes share several structural motifs, variation in these features produces distinct antigenic profiles (Figure 3). Interestingly, invasive GBS disease is most frequently associated with serotypes Ia, Ib, II, III, and V [59].

**FIGURE 3:**
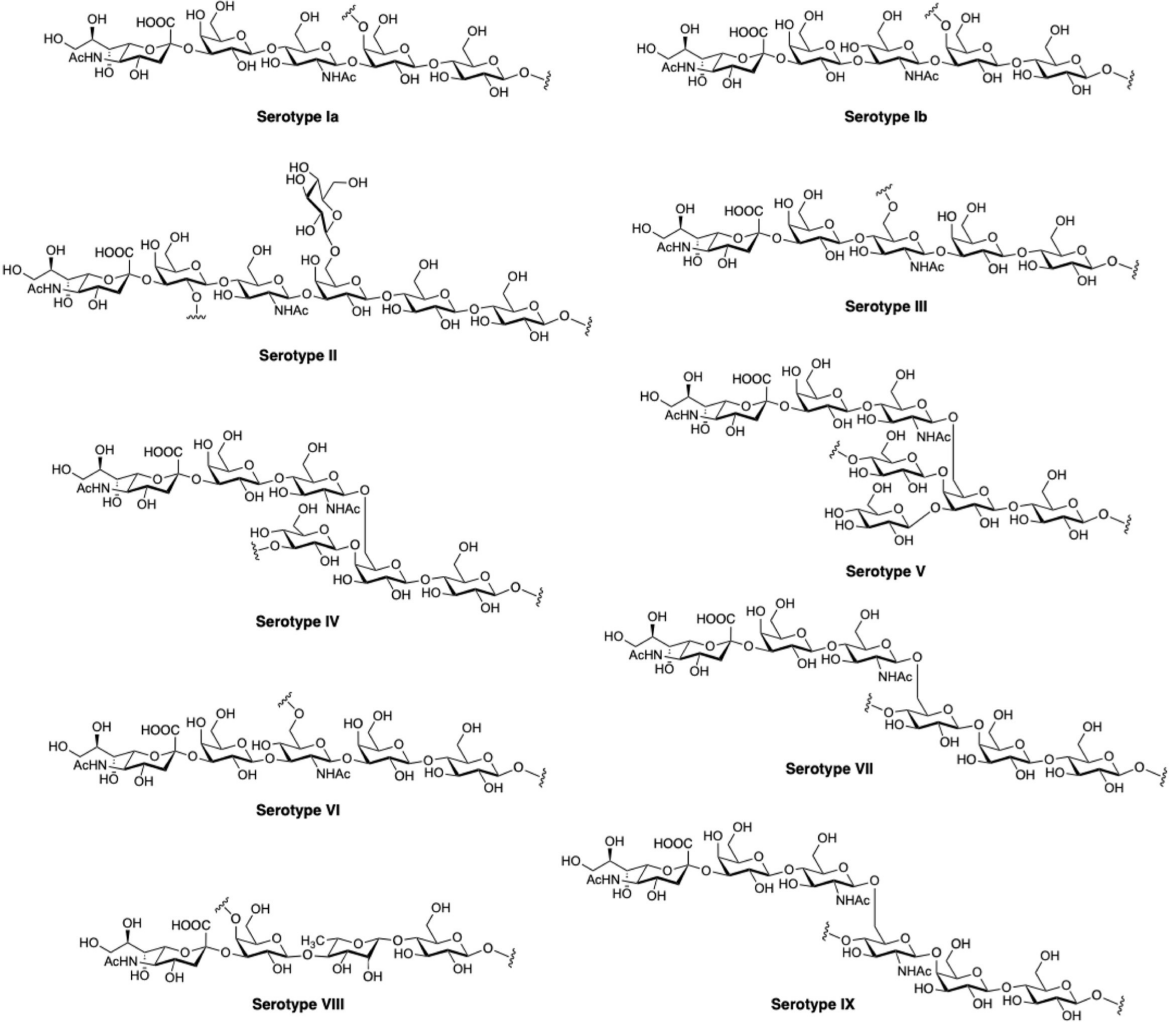
The chemical structures of GBS capsular polysaccharide repeating units.

## 5 Modes of Transmission of GBS

GBS is transmitted vertically, either in utero through ascending infection of the reproductive tract or during labor through direct contact between the newborn and vaginal bacteria [60, 61]. The mechanism by which GBS travels from the vagina to the fetus is currently poorly understood, primarily because GBS is non‐motile. After colonizing the rectovaginal tract through adhesin‐mediated attachment(Section 7.1), GBS must cross the cervical barrier, which provides both physical and chemical protection through its mucus plug and associated antimicrobial peptides (Section 8.1). GBS produces hyaluronate lyase, which degrades hyaluronic acid in host tissues and may facilitate bacterial movement through the reproductive tract [62] Upon reaching the lower uterus, GBS encounters the amniotic membranes. Its β‐hemolysin/cytolysin can damage host cells and has been implicated in membrane disruption, preterm premature rupture of membranes, and intrauterine infection [63]. GBS can subsequently cross the amniotic membrane and enter the amniotic fluid, placing the fetus at high risk (Figure 4).

**FIGURE 4:**
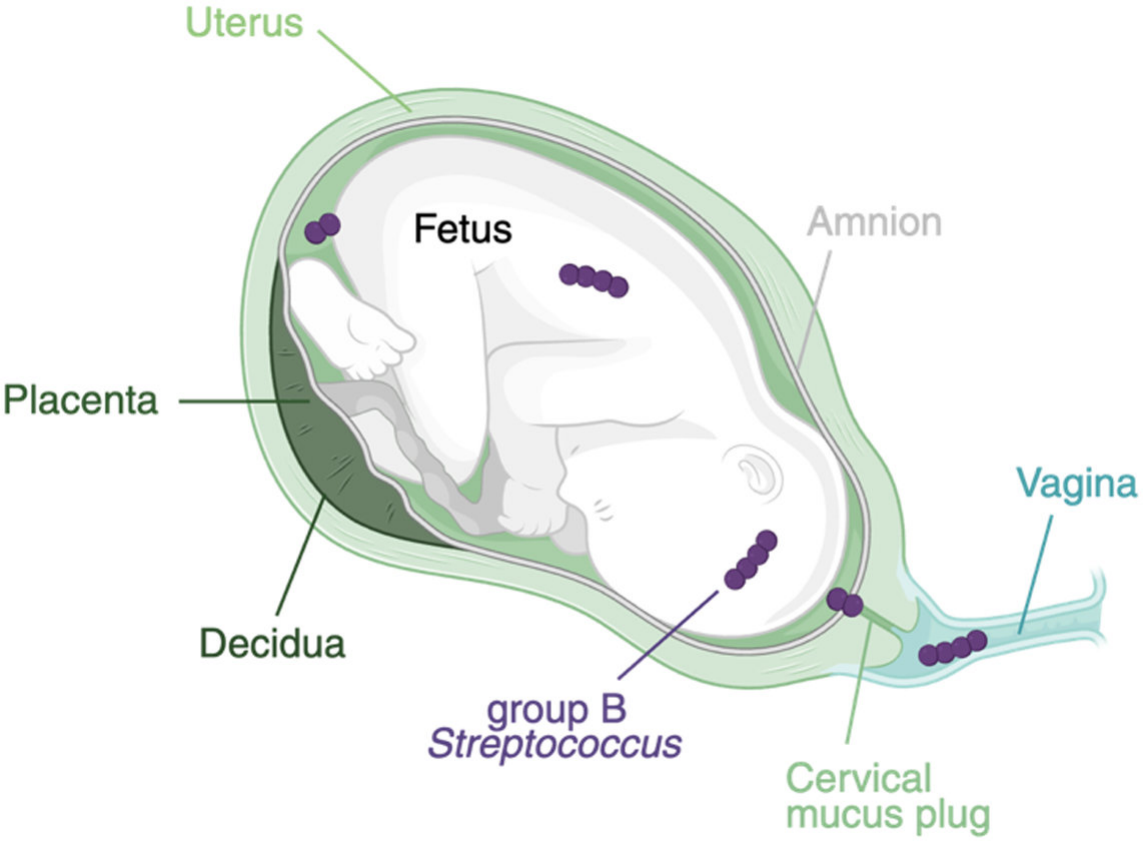
GBS ascends the reproductive tract first by vaginal colonization with movement through the cervical mucus plug, eventually crossing the amniotic membrane. The figure is colored to better illustrate structural features.

GBS can also be transmitted horizontally through contact with the mother, hospital personnel, or contaminated equipment [60]. Breast milk represents a third potential route of transmission; GBS has been detected in the milk of approximately 0.8%–3.5% of mothers [64, 65]. In one reported case, whole‐genome sequencing and phylogenetic analysis showed that a GBS strain isolated from the blood of a premature infant with late‐onset sepsis was genetically identical to strains recovered from the mother’s cervical secretions and breast milk [66]. These findings supported breast milk as the source of transmission in that case.

## 6 Clinical Burden of GBS Infection

GBS frequently colonizes the maternal gastrointestinal and urogenital tracts. Consequently, approximately 25% of pregnant women will test positive for GBS during pregnancy [67, 68]. While colonization rates remain steady among pregnant women globally, those in lower‐income regions, such as sub‐Saharan Africa and South Asia, face a higher risk of adverse pregnancy outcomes. These include chorioamnionitis, preterm birth (PTB), preterm prelabor rupture of membranes (PPROM), as well as early‐onset (EOD) and late‐onset disease (LOD), maternal sepsis, and both maternal and fetal death [69, 70, 71, 72].

### 6.1 Adverse Pregnancy Outcomes

Chorioamnionitis involves a sudden inflammation of the amniochorionic membranes and is associated with 40%–70% of preterm deliveries [26]. This condition poses serious risks to both the mother and infant, including postpartum infections and sepsis in the mother, and stillbirth, preterm birth, neonatal sepsis, chronic lung disease, and brain damage in the baby [14, 15, 16, 17, 18, 19, 20].

Premature rupture of membranes (PROM) occurs when the membranes break before labor begins. When this rupture happens before 37 weeks of gestation, it is called preterm PROM (PPROM), which accounts for about 3% of pregnancies and one‐third of all preterm births [27, 28, 29]. PPROM significantly contributes to perinatal mortality, mainly because it often triggers immediate labor after rupture. The severity of complications following PPROM largely depends on the gestational age at rupture but can worsen due to infections, placental abruption, or umbilical cord compression. Common issues associated with PPROM include respiratory syncytial virus (RSV), preterm birth, sepsis, necrotizing enterocolitis, and intraventricular hemorrhage [27, 28, 29].

PTB, or preterm birth, occurs when a baby is born before 37 weeks of pregnancy. In 2018, it was the second leading cause of infant mortality, and in 2020, approximately 10% of infants born in the U.S. were affected by PTB [35]. Racial and ethnic disparities are especially concerning, with the PTB rate among African American women reaching 13.8% in 2020, higher than the global rate of 12%. Risk factors include race, socioeconomic status, and maternal nutrition [36, 37, 38, 39, 40, 41]. The vaginal microbiome also plays a role; dominance of *L. crispatus* is linked to a lower risk of PTB [45, 46, 47, 48, 49]. PTB has lifelong consequences, including asthma, cerebral palsy, vision and hearing impairments, ADHD, and heightened anxiety [50, 51, 52].

### 6.2 Types of GBS Disease in Infants

GBS infections in newborns are categorized into three types depending on when the disease appears. Early‐onset disease (EOD) happens within the first 6 days of life, late‐onset disease (LOD) occurs from day 7 to 89, and very late‐onset disease (VLOD) takes place after 90 days (Table 3).

**TABLE 3:** Adverse pregnancy outcomes and manifestations that can occur due to GBS infection.

GBS disease in infants	Age	Mode of transmission	Manifestations	Mortality rate^a^
Early‐onset disease (EOD)	<7 days	Vertically	Sepsis, pneumonia, meningitis	2%–20%
Late‐onset disease (LOD)	7–89 days	Vertically or horizontally	Meningitis, sepsis, bacteremia	5%–15%
Very late‐onset disease (VLOD)	>90 days	Horizontally	Meningitis, sepsis, bacteremia	Variable

a Rates differ greatly due to the resources in high‐ versus low‐income countries.

EOD occurs within the first 6 days of life, usually presenting within 24 h. It is often caused by vertical transmission of bacterial pathogens from the mother during delivery or due to chorioamnionitis [73]. Serotypes Ia, III, and V account for 60%–70% of cases [64, 74]. EOD is associated with the highest infant mortality rate (~20%) because it can lead to pneumonia, bacteremia, sepsis, and meningitis [75]. LOD occurs between 7 and 89 days and is generally less deadly than EOD, mainly linked to serotype III [76]. In LOD, infection is usually acquired horizontally through skin‐to‐skin contact or infected breast milk [77, 78]. The most common and serious manifestation of LOD is bacterial meningitis [76], occurring in up to 50% of cases, along with bacteremia, septicemia, cellulitis, osteomyelitis, and lymphadenitis [78]. VLOD, the last GBS‐related infant disease, happens after 3 months of age. Though less common than EOD and LOD, its outcomes and risks are similar. Infants, especially those hospitalized for extended periods, are most at risk for VLOD through horizontal transmission.

## 7 GBS Virulence

GBS has various virulence factors that help it colonize the human host and cause infections (Table 4). This section discusses the general virulence factors involved in GBS colonization and immune evasion and highlights the factors that GBS uses to withstand the acidic environment of the vaginal niche.

**TABLE 4:** Key GBS adhesins and immune evasion factors.

Category	Factor	Function/mechanism
Anchoring	SrtA (sortase A)	Anchors LPXTG‐motif proteins to the cell wall
Adhesion	Pili	Hair‐like appendages for attachment and biofilm formation
	Srr1/Srr2	Glycosylated proteins for epithelial binding
	HvgA	Hypervirulent adhesin specific to serotype III
Invasion	PbsP	Binds plasminogen to assist endothelial invasion
	FbsA/FbsB/FbsC	Binds human fibrinogen
	C5a peptidase	Mediates binding to host tissue
Immune evasion	Capsular polysaccharide	Sialic acid mimicry, binds to host siglecs
	C5a peptidase	Cleaves and inactivates complement factors
Toxicity	β‐hemolysin	Pore‐forming cytolysin
	CAMP (Christie–Atkins–Munch‐Petersen) factor	Forms pores in host‐cell membrane

### 7.1 Adherence and Colonization

GBS relies on a variety of adhesins to establish colonization; however, these proteins must first be properly displayed on the cell surface. This anchoring is carried out by SrtA, a sortase that recognizes the LPXTG motif, the primary and best‐studied cell surface‐anchoring sequence in GBS [79]. Deletion of *srtA* produces significant defects in both adhesion and colonization, underscoring its foundational role in GBS invasion.

Several other proteins and structural features further enable GBS to establish a foothold in the host. Pili are non‐flagellar, externally expressed polymeric structures that facilitate adhesion, biofilm formation, and evasion of host immune responses [73, 80]. Two serine‐rich repeat proteins, Srr1 and Srr2, are transported via the SecA2 secretory system, during which they are heavily glycosylated by GtfA and GtfB [81]. This glycosylation is critical for successful attachment to and invasion across epithelial and endothelial barriers [82, 83, 84]. Similarly, three fibrinogen‐binding proteins, FbsA, FbsB, and FbsC, mediate attachment to human fibrinogen, a key blood‐clotting protein [85, 86, 87, 88]. PbsP, first identified in 2016, has since been shown to drive plasminogen‐dependent invasion of brain microvascular endothelial cells, contributing to meningitis during bacteremia [89]. More specifically, deletion of *pbsP* in a serotype III strain significantly reduced the bacterial burden, proinflammatory cytokine release, and mortality in a murine meningitis model [90]. The C5a peptidase, encoded by *scpB*, is most well‐known for its role in complement inactivation but has also been shown to facilitate host tissue adherence [91, 92, 93, 94]. Finally, HvgA was identified in 2010 through genomic analyses of hypervirulent serotype III isolates and is essential for GBS translocation across both the intestinal epithelium and the blood‐brain barrier [92, 93, 94, 95, 96].

### 7.2 Immune Evasion and Survival

For immune evasion, GBS relies primarily on its capsular polysaccharide (CPS). As discussed in Section 4.2, 10 known serotypes exist, all of which share a terminal sialic acid residue on their CPS side chain that is critical for pathogenesis [97, 98]. This sialic acid moiety enables the CPS to engage host sialic acid‐binding immunoglobulin‐like lectins (siglecs) on the surface of leukocytes, initially promoting bacterial adhesion [99]. More importantly, this sialic acid‐coated capsule effectively mimics host cell surface glycans, causing the immune system to recognize GBS as “self” and thereby dampening immune activation [100, 101].

Hemolysin production is another major contributing factor to GBS survival and virulence. Encoded by the *cyl* operon, this cytolysin doubles as a pigment responsible for the characteristic red/orange coloration of GBS colonies [102, 103]. The *β*‐hemolysin forms pores in host cells, including neutrophils and epithelial cells, causing direct cytotoxic damage [104, 105]. Interestingly, this lytic activity also carries immunosuppressive consequences; *β*‐hemolysin has been shown to trigger p38 MAPK‐dependent production of IL‐10, an anti‐inflammatory cytokine, effectively blunting the host inflammatory response [106]. The CAMP factor similarly initiates host cell lysis, facilitating intracellular invasion [107, 108]. Collectively, these strategies allow GBS to simultaneously evade immune surveillance and exert direct toxicity on host tissues.

### 7.3 Two‐Component Systems

Virulence factors are regulated in part by two‐component systems (TCSs), which enable bacteria to respond to environmental and cellular signals. A typical TCS consists of a membrane‐associated histidine kinase and a cytoplasmic response regulator. After detecting a stimulus, histidine kinase uses ATP to autophosphorylate a conserved histidine residue. It then transfers the phosphoryl group to a conserved aspartate residue in the receiver domain of the response regulator. This modification changes the regulator’s conformation and enables it to bind target promoters and alter gene expression. Depending on the system, the sensed signals may include changes in pH, nutrient availability, antibiotic exposure, temperature, osmolarity, membrane status, or intracellular heme levels [109].

Whole‐genome analyses indicate that GBS encodes approximately 17–22 TCSs [110, 111]. CovRS (control of virulence) is the best‐characterized system. It is conserved across the *Streptococcus* genus and regulates more than 100 genes, including genes involved in hemolysis, antimicrobial defense, adhesion, capsule‐associated functions, metabolism, and other aspects of virulence. CovRS can either activate or repress gene expression, and its effects vary among GBS strains and experimental models [112]. SaeRS has also been implicated in GBS pathogenesis [113, 114]. In *Staphylococcus aureus*, the SaeRS homolog acts as a central activator of virulence‐gene expression [115]. Evidence from GBS suggests a related function: SaeR activates *pbsP* expression (Section 7.2), and SaeRS is required for GBS proliferation in several animal models of invasive infection [114].

LytSR has also been implicated in GBS vaginal colonization and virulence. In a murine model, deletion of *lytS* reduced GBS persistence in the vagina, cervix, and uterus. The mutant also exhibited impaired biofilm formation, reduced autolysis, and decreased hemolytic activity. In a systemic infection model, loss of LytS attenuated bacterial dissemination and reduced the bacterial burden in both the blood and the brain. These findings indicate that LytSR contributes to both mucosal persistence and invasive disease, although the signals sensed by LytS and the direct targets of the LytSR regulon in GBS remain to be defined [116]. Given their broad regulatory effects, CovRS, SaeRS, and LytSR represent potential targets for therapeutic intervention.

### 7.4 Survival in the Vaginal Niche

The vaginal cavity presents a formidable challenge to most pathobionts as resident lactobacilli maintain a pH below 4.5 through the fermentation of glycogen into lactic acid. GBS, however, has evolved several mechanisms to withstand this acid stress. One such mechanism is alkali production via the arginine deiminase system (ADI), which converts arginine into ornithine, ammonia, and carbon dioxide while generating ATP. This ATP is then utilized to expel protons across the membrane, preserving cytoplasmic pH homeostasis [117, 118]. GBS also employs proton pumps for this purpose. F0F1‐ATPase is a multi‐subunit pump that promotes ATP synthesis during proton influx and, conversely, cleaves ATP to expel protons from the cell, thereby raising the cytoplasmic pH [118, 119, 120].

Beyond individual cellular defenses, GBS transitions from a planktonic state to a biofilm lifestyle to enhance environmental persistence [121, 122]. Biofilms are three‐dimensional communities that are encased in a self‐produced extracellular polymeric substance (EPS) matrix composed of proteins, extracellular DNA, and polysaccharides, which acts as both a physical and chemical shield [123, 124, 125]. The relationship between environmental acidity and GBS biofilm architecture remains contested in the literature, with evidence suggesting that biofilm formation is strain‐dependent or regulated by acidification [126, 127, 128]. This indicates that GBS strategically modulates its community structure in response to the individual vaginal niche.

In 2022, Thomas and Cook investigated *bvaP*, the most highly upregulated gene in the GBS strain A909 transcriptome when comparing vaginal colonization to growth in liquid culture [113, 129]. Deletion of *bvaP* produced altered cell morphology and hyperaggregation, and the gene was found to be essential for effective binding to certain ECM components and to human vaginal epithelial cells in vitro. Furthermore, *bvaP* was necessary for in vivo murine vaginal colonization. Collectively, these findings establish BvaP as a key contributor to GBS vaginal colonization and a promising candidate for therapeutic targeting.

## 8 Protection From and Treatment of GBS Infection

### 8.1 Host Protection: Maintenance of Asymptomatic Colonization

As a pathobiont, GBS must overcome a series of physical and chemical host barriers before transitioning from asymptomatic colonization to an invasive disease. The primary physical barrier is the vaginal mucosa, which is uniquely structured to trap and tolerate a degree of microbial presence while simultaneously limiting microbial adherence to the underlying epithelium. The cervical mucus plug (CMP) serves as an additional barrier, forming a thick, viscous cork within the cervix that physically obstructs bacterial ascent into the uterus [130, 131]. Chemically, CMP harbors antimicrobial components such as lactoferrin and lysozyme [132]. However, Rajagopal and colleagues found that the concentration of AMPs present in human CMPs was insufficient to directly kill GBS [131]. Instead, they found that CMP proteins activated leukocytes, resulting in elevated rates of bacterial killing, suggesting that CMP provides an indirect rather than direct mechanism for managing GBS abundance in the reproductive tract.

Vaginal epithelial cells also contribute to host defense through the production of AMPs, most prominently defensins and cathelicidins. The cationic nature of these peptides generates electrostatic attraction to the negatively charged GBS cell surface, enabling AMP‐mediated pore formation, leakage, and ultimately cell death [133, 134, 135]. GBS, however, has resistance to these AMPs through three mechanisms. First, GBS incorporates positively charged alanine residues into its cell wall teichoic acids, reducing the electrostatic affinity for cationic AMPs [136, 137]. Second, a specific penicillin‐binding protein, PBP1a, has been shown to confer increased resistance to both defensins and cathelicidins [138]. Third, the expression of the pilus backbone protein PilB is associated with improved resistance to cathelicidins and enhanced resistance to phagocytosis [73].

### 8.2 Antibiotic Use

Currently, antibiotics remain the primary intervention for GBS disease during gestation. Pregnant individuals who test positive for GBS during screening between 36 and 37 weeks of pregnancy are treated with intrapartum antibiotic prophylaxis (IAP) to prevent vertical transmission to the neonate [75, 139]. The choice of antibiotic depends on the susceptibility profile of the specific GBS strain and any documented maternal allergies (Figure 5).

**FIGURE 5:**
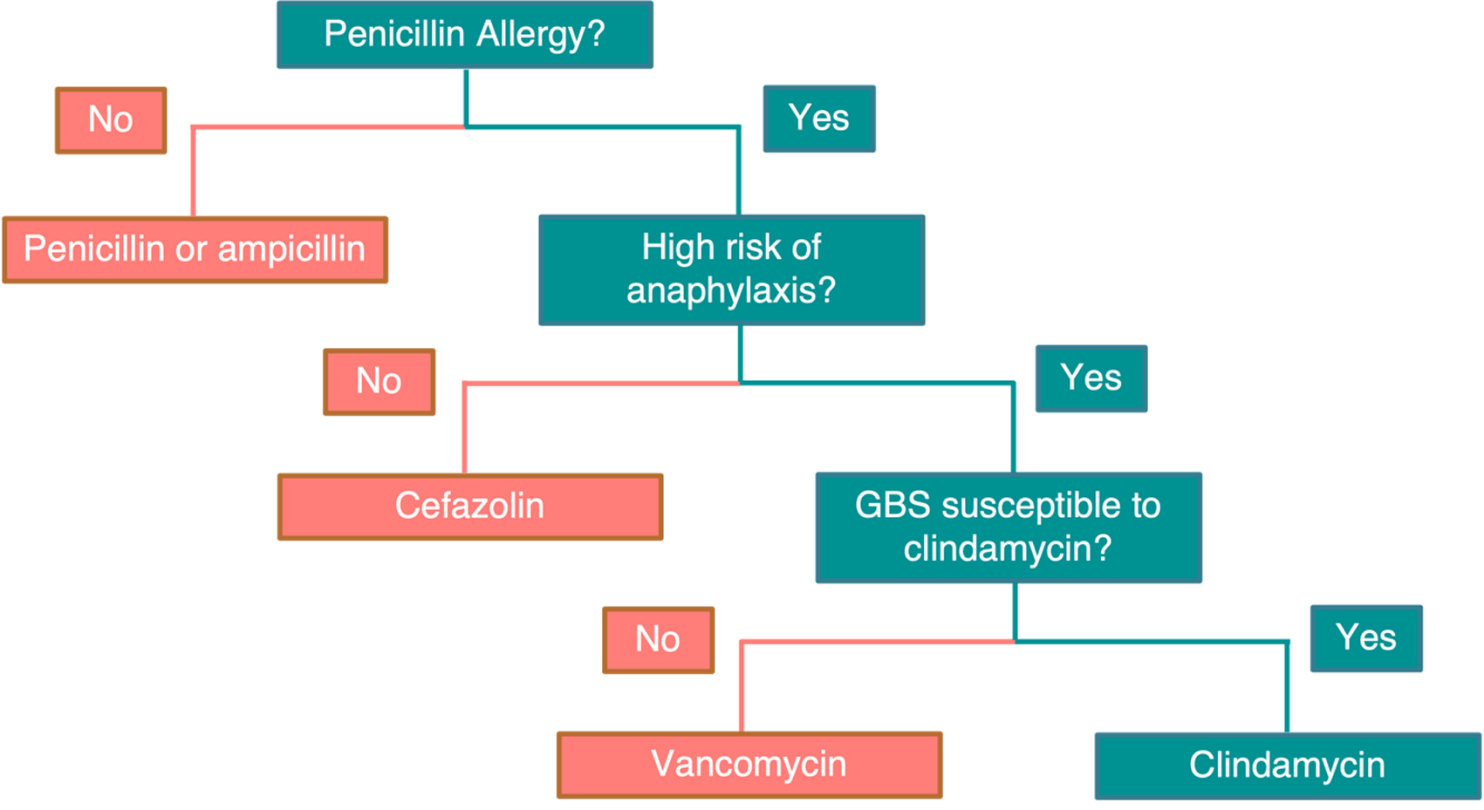
Guidelines for IAP treatment of GBS infections.

The preferred antibiotic class for GBS is *β*‐lactams, specifically penicillin and ampicillin. For the approximately 10% of women who report penicillin allergy, cefazolin, a first‐generation cephalosporin, is typically prescribed [140, 141]. Patients at high risk of anaphylaxis are given clindamycin if the GBS strain is susceptible [142]. Finally, vancomycin is given as a last‐resort antibiotic if the GBS strain is not susceptible to clindamycin. The structures of these antibiotics are shown in Figure 6A.

**FIGURE 6:**
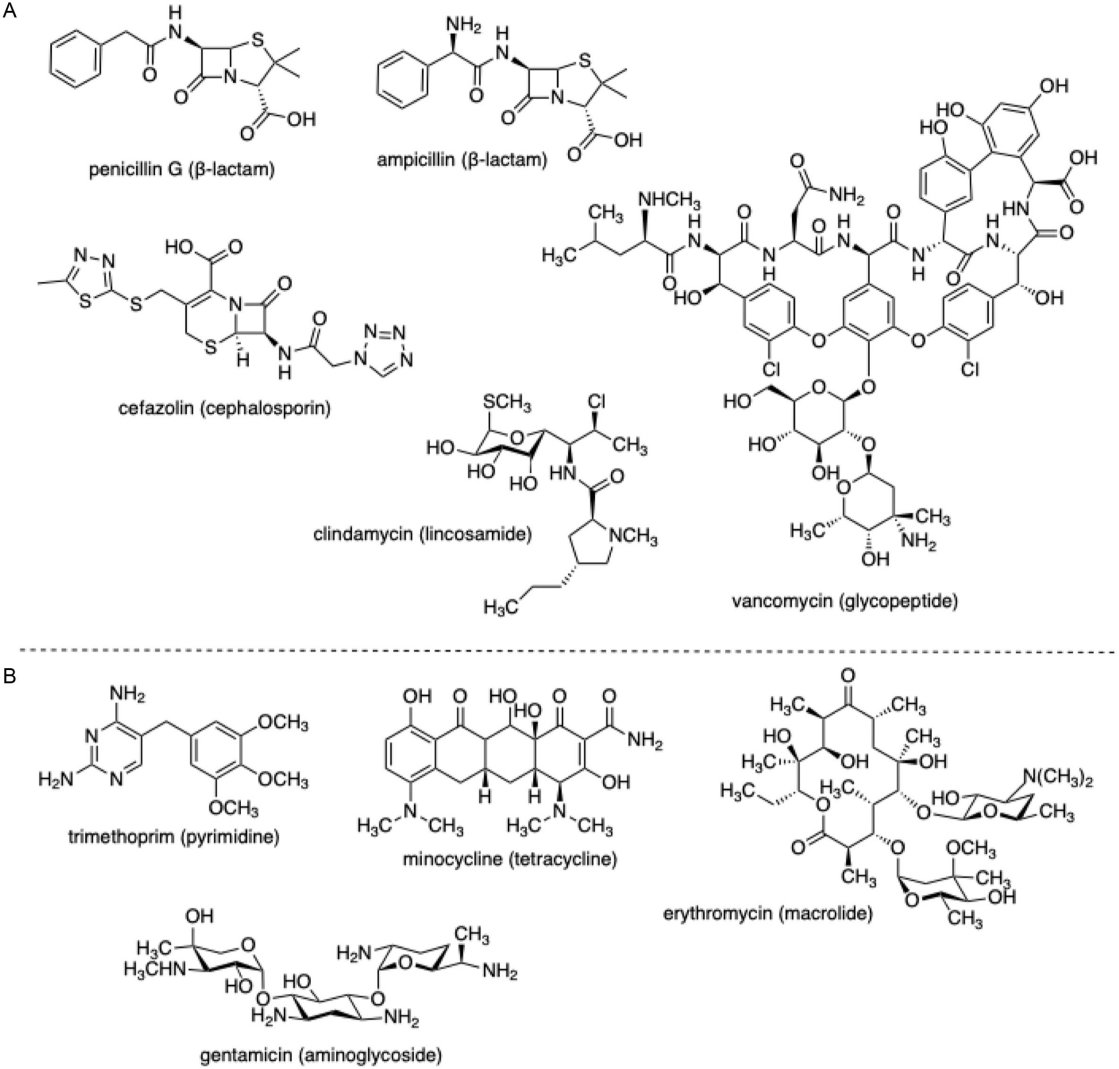
Commonly used antibiotics for GBS. (A) Antibiotics currently used in clinical practice and (B) examples of antibiotics to which GBS has resistance.

Since the discovery of penicillin approximately 100 years ago, researchers have worked to identify novel antibiotics, new bacterial targets, and strategies to circumvent resistance [143]. Antibiotic resistance occurs when bacteria develop the ability to withstand antibiotic treatment, rendering infections increasingly difficult to manage. The World Health Organization (WHO) found that 1 in 6 laboratory‐confirmed bacterial infections in 2023 were antibiotic‐resistant, highlighting the urgent need for novel therapeutics [144]. GBS either has intrinsic or acquired resistance to trimethoprim, erythromycin, minocycline, and gentamicin (Figure 6B), among others. The utility of antibiotics in this context is further complicated by their capacity to disrupt the nascent infant gut microbiota and their declining effectiveness in the face of emerging multidrug‐resistant strains [24, 25].

### 8.3 Vaccine Development for GBS

Vaccine research for GBS has expanded considerably over the past decade. Although no vaccine is currently approved, clinical trials through phase III have demonstrated promise for maternal immunization during the second or third trimester. Two leading candidates are currently under evaluation. Pfizer’s hexavalent capsular polysaccharide‐conjugate vaccine, GBS6, covers serotypes Ia, Ib, and II‐V and has recently advanced to phase III trials following encouraging phase II data [145, 147]. MinervaX’s surface‐protein‐based formulation, GBS‐NN/NN2, offers serotype‐independent protection and is currently in phase II trials [148, 149, 150, 151]. The CPS‐conjugate approach employed by GBS6 relies on a chemically defined coupling strategy essential for vaccine efficacy. GBS capsular polysaccharides undergo mild oxidation with sodium periodate, generating aldehydes on any sialic acid residue. These aldehydes are then irreversibly coupled to free amines on the carrier protein (tetanus toxoid or CRM197) through reductive amination, forming covalent CPS‐protein conjugates. This chemistry is critical because unconjugated CPS elicits only weak, T‍‐‍cell‐independent IgM responses, whereas PS‐protein conjugates promote robust T‍‐‍cell‐dependent IgG responses with enhanced opsonophagocytic killing capacity. The underlying strategy for both candidates is maternal immunization, which would confer passive immunity to the fetus through placental transfer of IgG antibodies capable of neutralizing GBS during early neonatal life.

### 8.4 Modulation of the Vaginal Microbiome With Prebiotics and Probiotics

An alternative approach to GBS mitigation involves modulating the vaginal microbiome through probiotic and prebiotic interven‐ tions. These strategies aim to establish or reinforce a Lactobacillus‐ dominant environment, either preconceptionally or during preg‐ nancy, to competitively exclude GBS. Clinical interest in these strategies is largely driven by observations that a high abundance of *L. crispatus* is negatively correlated with GBS colonization [152, 153, 154]. However, clinical trials utilizing *Lactobacillus* species as probiotics have produced inconsistent results, with discrepancies most commonly attributed to two factors: strain selection and administration route [155, 156, 157, 158]. Many studies employ probiotic strains originally isolated from the gastrointestinal tract, which may lack the niche‐specific adaptation factors required for long‐term vaginal persistence. Additionally, oral administration requires the probiotic to traverse the gastrointestinal tract prior to reaching the vaginal niche, a process that likely reduces the effective colonizing dose. Despite these challenges, several notable studies have demonstrated that targeted probiotic supplementation successfully reduced the GBS burden during gestation [159, 160]. However, we lack detailed mechanistic insights into how individual *Lactobacillus* strains and their metabolic products suppress GBS colonization in vivo; the relative contributions of lactic acid, H_2_O_2_, bacteriocins, and physical biofilm barriers remain incompletely quantified in the complex vaginal environment.

Prebiotic strategies take a complementary approach by providing selective substrates, such as host‐derived glycans, that preferentially support the growth of indigenous commensals while simultaneously inhibiting pathogen proliferation [161, 162, 163, 164, 165]. Vaginal prebiotic research has largely mirrored the inconsistencies seen in probiotic studies, primarily due to a lack of substrate specificity. Several studies have investigated generic plant‐derived oligosaccharides, which, while supportive of *Lactobacillus* growth, are also metabolized by pathogens. These findings underscore the need for vaginal‐niche‐tuned prebiotics capable of selectively promoting lactobacilli proliferation.

### 8.5 Limitations of Current Treatment Protocols

Despite the variety of strategies currently available, significant gaps remain in the global management of GBS. While IAP has successfully reduced the incidence of EOD by about 80%, it remains ineffective against LOD, PTB, or GBS‐induced stillbirth [72, 139, 166]. Furthermore, IAP is not a global standard as many low‐resource settings face substantial socioeconomic barriers to implementing universal screening and intravenous antibiotic administration [167]. Even in settings where IAP is available, rising rates of antimicrobial resistance and the well‐documented disruption of the nascent infant microbiome have intensified the search for non‐antibiotic alternatives.

Vaccine development has made meaningful progress, with lead candidates now advancing to phase III clinical trials. While early data suggest maternal receptivity to a GBS vaccine, societal challenges including vaccine hesitancy, particularly during pregnancy, and the logistical complexities of global distribution remain significant hurdles [168, 169]. Similarly, probiotic and prebiotic interventions offer a promising microbiome‐friendly approach, but clinical results have been inconsistent and standardized, evidence‐based protocols have yet to be established. Collectively, these persistent gaps in prevention and treatment underscore the urgent need for novel, targeted strategies capable of modulating GBS colonization without the collateral damage associated with conventional antibiotic use.

## 9 Outlook

Despite recent advances in microbiome characterization, significant gaps remain in our understanding of vaginal microbial function and GBS pathogenesis. While metagenomics and metatranscriptomics have revealed the taxonomic composition of CSTs and identified functional gene expression patterns, we lack detailed mechanistic insights into how individual *Lactobacillus* strains and their metabolic products suppress GBS colonization in vivo. For example, the relative contributions of lactic acid, H_2_O_2_, bacteriocins, and physical biofilm barriers remain incompletely quantified in the complex vaginal environment. Furthermore, the drivers of individual variation in susceptibility to GBS colonization and progression to infection remain poorly understood; while genetics, ethnicity, and socioeconomic factors shape the microbiome composition, the mechanisms linking these variables to clinical outcomes require deeper investigation. Future studies employing longitudinal sampling, refined genomic tools, and mechanistic validation in physiologically relevant models will be essential to translate microbiota‐level observations into predictive biomarkers and targeted interventions.

The limitations of intrapartum antibiotic prophylaxis and its ineffectiveness against late‐onset disease, preterm birth, and stillbirth, combined with growing antimicrobial resistance concerns, have catalyzed interest in alternative prevention strategies. Probiotic and prebiotic interventions represent promising avenues, yet current formulations remain largely untargeted and inadequately tested in rigorous clinical trials. Future directions should prioritize the development of niche‐tuned therapeutics: consortia or single‐strain probiotics specifically engineered to establish or reinforce CST I dominance, coupled with prebiotics that selectively fuel *Lactobacillus* metabolism while starving pathobionts. Additionally, strain‐level precision and personalized microbiome assessment may enable risk stratification, identifying pregnant individuals with dysbiotic or GBS‐permissive microbiota who would most benefit from preconception or antenatal microbiota modulation. Such precision approaches could minimize unnecessary intervention while maximizing efficacy in high‐risk populations.

The documented disparities in CST distribution across ethnic and socioeconomic groups underscore the urgent need for equity‐centered research in vaginal microbiome science. Future investigations must interrogate not only the biological basis of these differences but also their social determinants, including differential antibiotic exposure, healthcare access, and contraceptive use patterns, to develop interventions that address root causes rather than symptoms. Translating microbiota‐targeting strategies from bench to bedside will require large‐scale, geographically and ethnically diverse clinical trials, integrated microbiome monitoring in obstetric practice, and stakeholder engagement to ensure that novel therapies are accessible and culturally appropriate. Ultimately, realizing the therapeutic potential of vaginal microbiota modulation demands interdisciplinary collaboration spanning microbiology, immunology, obstetrics, and public health, with the goal of reducing the burden of GBS‐associated morbidity and mortality globally, particularly in underserved populations.

## Funding

This work was supported by the National Institutes of Health under Grant no. R35‐GM133602.

## Conflicts of Interest

The authors declare no conflicts of interest.

## Data Availability

Data sharing not applicable to this article as no datasets were generated or analysed during the current study.
